# Spent Coffee Grounds as an Adsorbent Material for Metal Ions

**DOI:** 10.3390/ma19091720

**Published:** 2026-04-23

**Authors:** Krystyna Pyrzynska

**Affiliations:** Faculty of Chemistry, University of Warsaw, 02-093 Warsaw, Poland; kryspyrz@chem.uw.edu.pl

**Keywords:** spent coffee grounds, modification, adsorption, metal ions, waste treatment

## Abstract

The valorization of agricultural and food industry residues represents an important component of the circular bioeconomy, enabling the conversion of waste streams into value-added materials while mitigating environmental pollution. Spent coffee grounds (SCGs), a solid by-product generated during the extraction of coffee beans with hot water or steam, constitute an abundant lignocellulosic biomass residue. Due to their physicochemical properties, SCGs can be used as low-cost adsorbent materials for the treatment of metal-contaminated wastewater, offering a sustainable alternative to traditional synthetic resins. This review summarizes recent research on the application of SCGs for the removal of metal ions from aqueous systems. The adsorption performance of raw and modified SCGs, including materials obtained via carbonization and chemical functionalization, is comparatively evaluated. Furthermore, key operational parameters governing the adsorption process and the corresponding metal removal efficiencies are discussed.

## 1. Introduction

The presence of metal ions in aquatic environments, originating from both natural processes and anthropogenic activities such as mining, industrial discharge, and agriculture, represents a significant environmental and public health concern [1]. Certain metals, including mercury, cadmium, chromium, and lead, have attracted particular attention due to their high toxicity. Permissible limits for these metals in wastewater discharge vary depending on regulatory frameworks, including those established by the World Health Organization, the Environmental Protection Agency, and national authorities, as well as the type of industry. Generally, stricter standards apply to direct discharge into natural water bodies compared with discharge into sewer systems. These limits are typically set within the µg/L to low mg/L range, depending on the specific metal and regulatory context. Consequently, wastewater containing heavy metals must be treated prior to discharge.

Numerous treatment technologies have been investigated for metal ion removal, including chemical precipitation, ion exchange, membrane filtration, electrochemical deposition, and coagulation–flocculation processes. More advanced approaches include membrane bioreactors, advanced oxidation/reduction processes, forward osmosis, and electrocoagulation. Although these techniques can achieve high removal efficiencies, they often present significant drawbacks, such as high operational costs, sludge generation, secondary pollution, and complex maintenance requirements. For example, membrane technologies frequently suffer from fouling and scaling, while advanced oxidation processes are associated with high energy and chemical consumption. These methods have been extensively reviewed elsewhere [2,3]. Compared with alternative treatment approaches, adsorption is widely regarded as one of the most promising techniques due to its operational simplicity, high efficiency, and potential for adsorbent regeneration and reuse [4,5,6].

In recent years, increasing attention has been directed toward low-cost and environmentally friendly adsorbents derived from agricultural and food wastes, often referred to as “green adsorbents” [7,8]. Spent coffee grounds (SCGs), a major by-product of global coffee consumption, have emerged as a promising precursor for the development of such materials [9]. SCGs are readily available in large quantities, with approximately 650 kg generated per ton of processed coffee beans [10]. In the production of soluble coffee, roughly 2 kg of wet SCGs are produced per kilogram of product [11]. These wastes are typically disposed of in landfills, which can lead to environmental issues, including emissions of methane and carbon dioxide, as well as the leaching of organic compounds into soil and water systems.

The chemical composition of SCGs is dominated by polysaccharides (cellulose, hemicellulose, and lignin), lipids, nitrogenous compounds, and phenolic substances [12,13,14]. Owing to this composition, SCGs have demonstrated considerable potential for valorization into functional food ingredients [15,16], cosmetic [17,18], and biodegradable packaging materials [19]. Furthermore, sequential extraction processes enable the recovery of valuable compounds for the production of biodiesel, bioethanol, and composite materials [20,21,22]. Due to their porous lignocellulosic structure, relatively high surface area, and abundance of surface functional groups, SCGs are effective in removing dyes, pesticides, and metal ions from contaminated water [23,24,25]. Moreover, SCGs can be converted into high-surface-area biochar via pyrolysis, typically followed by chemical activation, yielding porous carbon materials suitable for water treatment and soil amendment [26,27,28].

Thus, the valorization of SCGs as an adsorbent supports both wastewater treatment and waste management within the framework of the circular economy. Despite the growing number of studies on SCG-based adsorbents, their systematic and quantitatively comparable evaluation remains limited [29]. This review summarizes recent advances in the adsorption performance of raw and modified SCGs, including carbonized and functionalized materials for the removal of metal ions from aqueous systems. In addition, key parameters influencing adsorption processes and the corresponding removal efficiencies are discussed.

## 2. Literature Search Methodology

To provide a comprehensive and structured overview of current research on SCGs as adsorbents for metal ion removal, a systematic literature search was conducted using the Scopus, Web of Science, and Google Scholar databases. The search primarily covered studies published between 2010 and 2024, with particular emphasis on developments from the last decade. The following keywords and their combinations were used: spent coffee grounds, SCG, adsorption, biosorption, metal ions, biochar, modification, and wastewater treatment.

The inclusion criteria comprised studies investigating SCGs or SCG-derived materials for metal ion removal from aqueous solutions, experimental studies reporting adsorption performance (e.g., adsorption capacity, removal efficiency), and studies providing information on material preparation, modification, or characterization. The exclusion criteria included studies focusing exclusively on organic pollutants (e.g., dyes, pharmaceuticals) and studies lacking sufficient experimental detail.

For the comparative analysis only studies reporting quantitative adsorption data under clearly defined experimental conditions were considered. When multiple datasets were available, representative values were selected based on completeness of reported parameters, including pH, adsorbent dosage, and contact time. Despite these criteria, variability in experimental conditions across studies remains a significant limitation, which is critically discussed in Section 4.

## 3. SCG-Based Adsorbents

**Figure 1:**
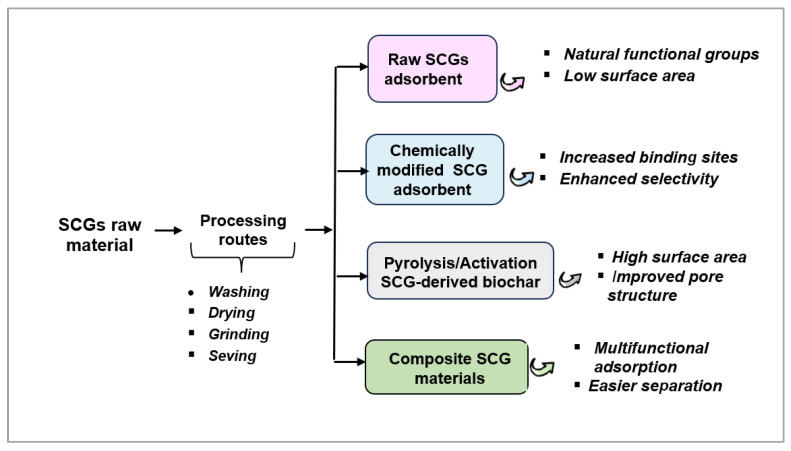
Preparation pathways and modification strategies for SCG-based adsorbents.

Spent coffee grounds represent a versatile precursor for a wide range of adsorbent materials, from raw biomass to highly engineered carbon-based structures. The preparation pathways and modification strategies for SCG-based adsorbents are shown in Figure 1. Depending on the preparation method and degree of modification, SCG-based adsorbents exhibit significantly different physicochemical properties and adsorption performance.

These materials have been extensively characterized using a combination of microscopic and spectroscopic techniques [30]. Techniques such as Fourier-transform infrared spectroscopy (FTIR) and ^13^C NMR are used to identify surface functional groups and carbon structure, while X-ray photoelectron spectroscopy (XPS) provides information on surface elemental composition and oxidation states. The Boehm titration is commonly employed to quantify acidic surface groups.

Morphological characteristics, including particle size, shape, and aggregation, are typically analyzed using scanning electron microscopy (SEM), transmission electron microscopy (TEM), and dynamic light scattering (DLS). X-ray diffraction (XRD) is used to assess structural properties, while thermogravimetric analysis (TGA) provides insight into thermal stability and decomposition behavior. Surface area and pore structure are determined using BET nitrogen adsorption–desorption isotherms. Additionally, surface charge is commonly evaluated by determining the point of zero charge (pH_p_zc).

### 3.1. Raw SCG Adsorbents

Raw SCGs exhibit an irregular and porous structure, with particle size depending on the brewing method and initial grind size. Typically, particle sizes range from a few micrometers to approximately 2 mm, with most particles in the 100–1000 µm range [30].

SCGs are usually dried to remove moisture and prevent microbial degradation. Washing with deionized water is commonly performed to remove residual soluble compounds, such as caffeine and polyphenols, which may interfere with adsorption. FTIR analysis confirms that raw SCGs are rich in oxygen-containing functional groups, including hydroxyl, carbonyl, and ester groups, which facilitate metal ion binding. XRD analysis indicates a predominantly amorphous structure characteristic of lignocellulosic materials.

SCGs are rich in hemicellulose (30–40%) and cellulose (8–15%) as well as proteins (13–17%), lignin (20–30%), and lipids (7–21%) [31,32,33]. According to Mussato et al. [34], SCG contains 46.8% mannose, 30.4% galactose, 19% glucose, and 3.8% arabinose. The main fatty acids in the SCG oil fraction are linoleic, palmitic, oleic, and stearic. Chlorogenic and caffeic acids are the main phenolic compounds [21,35]. Some flavonoids, such as quercetin, rutin, and cyanidin-3-glucoside, have also been reported [36]. Polysaccharides, proteins, and phenolic compounds contribute to the abundance of oxygen-containing functional groups, which serve as binding sites for metal ions. The chemical composition of SCGs is influenced by coffee species, cultivation conditions, roasting, and brewing methods [37]. For example, robusta contains more caffeine and chlorogenic acids, whereas arabica is richer in lipids and sucrose. Environmental factors such as climate, soil composition, and altitude also affect coffee bean chemistry, which is subsequently reflected in SCG compositions [38].

Several studies have demonstrated that raw SCGs can simultaneously adsorb and reduce Cr(VI) to Cr(III) [39,40]. Structural characterization and FTIR analysis indicated that HCrO_4_^−^ ions are initially adsorbed onto the surface of SCGs via hydrogen bonding, subsequently reduced to Cr(III) by electron-donating phenolic compounds and then in situ co-precipitated or complexed with iron oxyhydroxides.

### 3.2. Chemically Modified SCG Adsorbents

**Figure 2:**
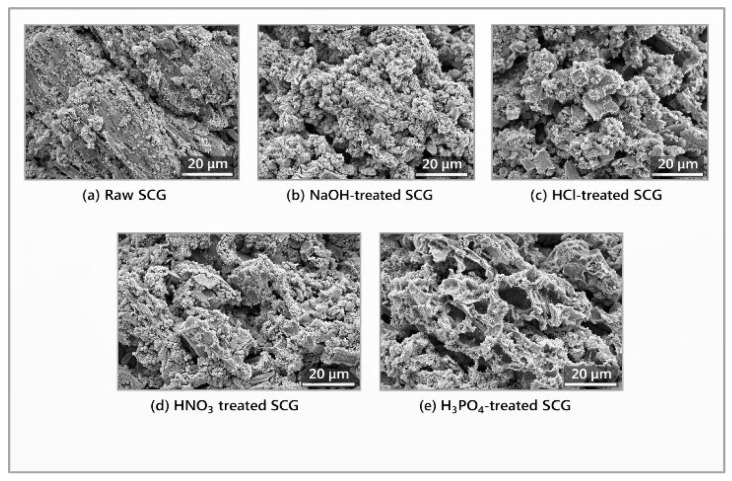
Comparative SEM morphology of raw and chemically modified SCG. Own compilation based on literature data [42,43,44,45,46].

Chemical treatment of SCGs using acids or bases enhances adsorption performance by increasing porosity and introducing additional functional groups, such as carboxyl and hydroxyl groups [41,42]. Comparative SEM micrographs of raw and chemically modified SCGs are presented in Figure 2. Raw SCGs exhibit a sponge-like porous structure (Figure 2a). After NaOH treatment, the material shows fragmented particles and increased surface roughness (Figure 2b). HCl modification results in surface cleaning and partial pore opening (Figure 2c). In contrast, HNO_3_ treatment produces an oxidized and etched morphology (Figure 2d), whereas H_3_PO_4_-modified SCGs display visible cracks and a more developed porous structure (Figure 2e).

Raw SCGs were also modified with calcium alginate for the removal of copper ions from water [47]. The resulting material combines the high adsorption kinetics of SCGs with the structural stability and binding capacity of calcium alginate. Treatment with natural deep eutectic solvents (NaDES) helps break down the lignocellulosic structure of SCGs, exposing more binding sites for metal ions [48]. It has been shown that using a choline chloride and urea/citric acid mixture increases Pb(II) adsorption to 35.95 mg/g compared with untreated SCGs (21.53 mg/g). Pretreatment with ionic liquids (ILs) also increases the material’s surface reactivity, making it more susceptible to further activation processes [49]. Although ILs and NaDES are effective, their environmental impact and recyclability remain important considerations.

A significant improvement in adsorption capacity compared with raw coffee waste was obtained using KMnO_4_-modified SCGs for Pb(II) [50] and Cd(II) [51] removal. KMnO_4_ deposits manganese oxides (MnOx) on the surface, additionally enhancing metal ion binding through a surface complexation with MnOx and oxygen-containing functional groups.

Coating SCGs with iron precursors via mechanical mixing, wet impregnation, or precipitation techniques is an effective modification strategy [52,53,54]. The intrinsic polyphenolic compounds present in SCGs act as reducing agents, converting iron ions into magnetic iron species such as zero-valent iron or iron oxide nanoparticles, without the need for elevated temperatures. This approach enables facile magnetic separation of the modified adsorbents from aqueous media after treatment.

The effectiveness of chemical modification depends strongly on process parameters such as reagent concentration, treatment temperature, and reaction time, which directly affect pore development and surface functionality. SCG-modified materials enhance adsorption performance through multiple mechanisms, including electrostatic interactions, pore filling, and π–π interactions, compared with raw SCGs. Although chemically modified SCGs generally exhibit improved performance compared with raw materials, their production requires additional reagents and processing steps, which may increase cost and environmental impact.

### 3.3. SCG-Derived Biochar

To overcome the limitations of raw SCGs, such as low structural stability and limited porosity, increasing attention has been directed toward producing SCG-derived biochar. Biochar is typically produced via pyrolysis under oxygen-limited conditions at temperatures ranging from 300 to 850 °C [27,55,56,57]. Lower pyrolysis temperatures tend to preserve functional groups, whereas higher temperatures favor pore development. For example, SCG-derived biochar produced at 300 °C exhibited superior performance in the preconcentration of Pb(II) compared with biochars prepared at temperatures up to 700 °C [58]. Although higher pyrolysis temperatures generally increase surface area, they simultaneously reduce biochar yield and may destroy surface functional groups. In some approaches, torrefaction at lower temperatures (200–300 °C) has been used as a preliminary pretreatment step before pyrolysis [59,60]. Torrefaction reduces bulk density, while increasing surface area and pore volume [59]. Overall, biochar adsorption performance depends on a balance between surface area and the availability of functional groups.

Enhancement of pore structure and surface properties is commonly achieved through subsequent activation processes, including physical activation with CO_2_ or N_2_, as well as chemical activation or modification using KOH, H_3_PO_4_, ZnCl_2_, or FeCl_3_ [13,27,28]. While chemical activation generally yields higher surface areas, it may involve the use of corrosive reagents. The primary objective of activation is to develop a well-defined pore structure, whereas modification aims to tailor surface chemistry by introducing additional functional groups.

Magnetic biochar derived from spent coffee grounds is typically prepared by impregnating or synthesizing iron species onto the biochar surface or within its pore structure [61,62]. Fe-modified SCG biochar produced under a CO_2_ atmosphere exhibited higher maximum magnetization than that synthesized under N_2_ [63]. However, the stability of iron species on the biochar surface and potential iron leaching, particularly under acidic conditions, may result in effluent concentrations.

Modification of SCG-based biochar with complexing ligands is an effective strategy to enhance metal-ion adsorption capacity. Sulfur functionalization, often achieved through sulfhydryl (thiol) groups, transforms biochar into a soft Lewis base, highly effective at targeting Cd(II), Hg(II), and Pb(II) [64,65,66,67]. Amine groups introduced onto the biochar surface can act as hard/intermediate Lewis bases, thereby improving the adsorption of acidic pollutants and heavy metals [68,69]. Some modifications use compounds containing both sulfur and nitrogen, such as isothiocyanates, which provide selectivity for a wider range of both hard and soft acid metal ions [67]. The results indicated that the removal of metal ions by modified-biochars follows the order Pb(II) > Cr(III) > Cd(II) ~ Ni(II) and Cd(II) > Pb(II) > Ni(II) ~ Cr(III) for mono-element and multi-element systems, respectively.

**Figure 3:**
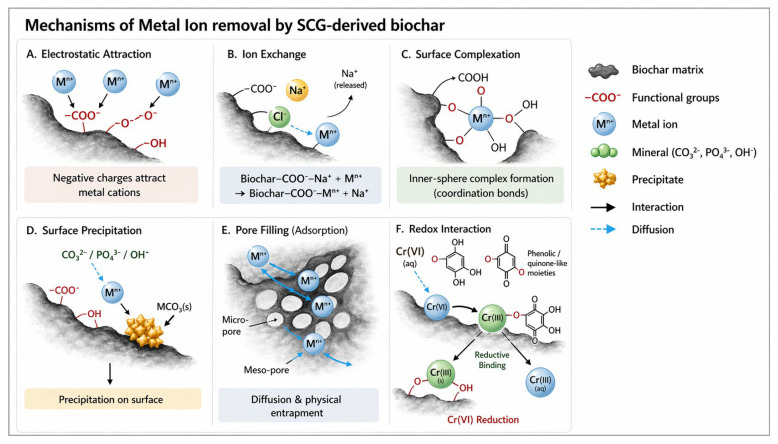
The main mechanisms for the removal of metal ions by SCG-derived biochar.

Table 1 summarizes representative pyrolysis and activation/modification conditions used to convert SCGs into carbonaceous adsorbents for metal-ion removal. Figure 3 presents different mechanisms utilized for metal ion removal by SGC-derived biochars. Their adsorption performance and operating conditions will be discussed in Section 4.

The reviewed studies indicate that SCG-derived adsorbents for metal-ion removal are predominantly produced via slow pyrolysis at 400–700 °C, most commonly within the 500–600 °C range, using moderate heating rates (5–10 °C/min) and residence times of 1–2 h under a N_2_ atmosphere. A slow heating rate promotes more uniform carbonization. Unmodified biochars generally exhibit low specific surface areas (<50 m^2^/g), whereas chemical activation, particularly with KOH or H_3_PO_4_, substantially enhances porosity, yielding surface areas from approximately 350 to over 1600 m^2^/g [75]. Iron-based modifications introduce additional redox and magnetic functionalities but do not necessarily yield very high surface areas, indicating that adsorption in these systems is partly governed by surface complexation and redox interactions.

Overall, adsorption performance depends not only on pyrolysis temperature but, more importantly, on activation strategy and resulting surface chemistry. Lower temperatures favor preservation of functional groups, whereas chemical activation is the most effective approach for maximizing surface area and metal-ion uptake.

### 3.4. SCG-Composite Materials

The integration of inorganic nanoparticles, polymers, or mineral phases into SCG-derived matrices facilitates the development of multifunctional adsorbents exhibiting enhanced adsorption capacity, superior structural integrity, and precisely tailored surface functionality [81]. Moreover, SCG-composite materials are particularly promising for selective metal-ion sequestration, as they enable controlled functionalization of surface groups and the incorporation of metal-specific coordination sites.

Chitosan, a natural polycationic polymer, is often combined with SCGs to create beads or aerogels [82,83,84]. The composite aerogel, synthesized in a one-pot process from chitosan, cellulose, FeCl_3_, and a coffee extract solution, enhanced arsenic adsorption at pH 3–7 via electrostatic interactions with chitosan amine functional groups and complexation/coordination with the incorporated iron particles [82]. For As(V), a much higher maximum adsorption capacity (22.57 mg/g) was obtained than for As(III) (2.27 mg/g). UV crosslinking composite containing phenyl azide grafted onto chitosan and subsequently mixed with SCGs was proposed by Zheng and Chien [83]. The adsorption capacity of the proposed composite for Cu(II) was nearly twice that of chitosan alone. Magnetic composite, synthesized using activated carbon from SCGs, chitosan from shrimp shells, and crosslinked with green tea extract, achieved a maximum Ni(II) adsorption capacity of 108.70 mg/g [84]. This composite maintained a high removal efficiency of over 86% after five consecutive adsorption/desorption cycles.

Spent coffee grounds modified with polyethyleneimine (PEI) were used for the successive removal of AsO_4_^3−^, Cu^2+^, and PO_4_^3−^ ions, exploiting changes in the net surface charge after the adsorption of each ion [85]. The SCGs were treated with PEI, cross-linked with glutaraldehyde, and subsequently loaded with Fe(III). The amino groups of PEI initially provide a positive surface charge due to protonation, while Cu(II) ions are removed primarily through strong metal coordination with nitrogen lone pairs. Subsequent adsorption of arsenic or phosphate ions occurs via ligand exchange, in which hydroxyl groups coordinated to Fe(III) sites are replaced. This sequential adsorption process utilizes the versatile functional groups of the SCG–PEI–Fe composite.

SCG-derived hydrogels were synthesized by incorporating humic-like substances (extracted by 0.1 M NaOH solution from SCGs) into a polyacrylic acid network via one-step radical polymerization [86]. These hydrogels exhibit high sorption capacities for U(VI) under acidic conditions and strong resistance to interfering ions. The removal process involves monolayer chemisorption, in which U(VI) ions interact with carboxyl and hydroxyl groups on the hydrogel surface.

Le et al. [87] proposed a composite for the removal of Pb(II) prepared by integrating Fe_3_O_4_ nanoparticles, polyvinyl alcohol (PVA), and alkaline-pretreated SCGs. PVA helps bind the components and provides hydroxyl groups that can interact with lead ions, while Fe_3_O_4_ imparts magnetic properties for easy separation after adsorption. SCGs serve as a biomass scaffold offering a carbonaceous porous structure and additional surface functional groups.

MgO carbon-based composite was prepared by hydrothermal synthesis of MgO nanoparticles and magnetic SCG-biochar to remove Pb(II) from aqueous solution [88]. The main adsorption mechanism was surface precipitation, PbO and Pb_3_(CO_3_)_2_(OH)_2_, accompanied by the formation of complexes, such as R-COO-Pb and R-O-Pb. The maximum Pb^2+^ sorption capacity of 321.9 mg/g was significantly higher than that of magnetic biochar alone (14.1 mg/g).

A multifunctional strategy for Cr(VI) remediation was reported by Ly et al. [89] based on a magnetic biochar composite integrating molybdenum disulfide and silver nanoparticles. In this system, biochar derived from SCGs serves as an adsorptive and conductive scaffold, concentrating Cr(VI) species at the interface and facilitating electron transfer. MoS_2_ provides catalytically active sulfur edge sites that promote reductive transformation of Cr(VI) to Cr(III). At the same time, Ag nanoparticles act as electron mediators and sinks, enhancing interfacial charge transfer and suppressing recombination. The reduced Cr(III) species are subsequently immobilized via surface complexation or precipitation, thereby minimizing the risk of secondary contamination. The incorporation of the microalga *Spirulina maxima* introduces an additional bio-assisted component, primarily through biosorption and redox-active functional groups, thereby complementing the inorganic catalytic framework. Collectively, this hybrid design exemplifies the integration of adsorption, catalysis, photocatalysis, and biogenic functionalities within a single remediation platform. However, the synthesis of that composite is quite complex, and its stability and long-term performance under repeated use need thorough testing.

## 4. SCG-Based Adsorbents for the Removal of Metal Ions

Table 2 summarizes the adsorption capacity (q_max_) of different SCG-based materials reported in the literature for the removal of various metal ions. Adsorption capacity is one of the most frequently reported parameters describing adsorbent performance, reflecting a material’s ability to uptake specific metal ions and enabling comparison with other sorbents. In addition, key experimental parameters influencing adsorption performance, such as solution pH, the ratio of adsorbent dose to sample volume, and contact time, are also presented.

### 4.1. Comparison of Adsorption Capacity and Efficiency

The adsorption capacities summarized in Table 2 demonstrate the wide range of SCG-based materials developed for the removal of various metal ions. However, direct comparison of reported q_max_ values across studies remains challenging due to significant variability in experimental conditions, including solution pH, initial metal concentration, adsorbent dosage, and contact time. To address this limitation, a comparative analysis should consider not only the reported adsorption capacity but also the corresponding experimental conditions. In particular, pH plays a critical role in controlling both metal speciation and adsorbent surface charge, while adsorbent dosage and initial concentration strongly influence the apparent adsorption capacity due to site saturation effects. Therefore, comparisons are most meaningful when studies are conducted under similar conditions or when trends are evaluated within the same class of materials.

Based on the reviewed literature, SCG-based adsorbents can be broadly categorized into four groups: raw SCGs, chemically modified SCGs, SCG-derived biochars, and composite materials, as shown in Figure 1. Each group exhibits distinct adsorption behavior and performance characteristics. Raw SCGs generally exhibit moderate adsorption capacities, typically below 50 mg/g for most metal ions, which can be attributed to the presence of naturally occurring functional groups such as hydroxyl, carboxyl, and phenolic moieties. However, their relatively low surface area and limited accessibility of active sites constrain their overall performance.

Chemical modification significantly enhances adsorption capacity by increasing the number and accessibility of functional groups. For example, oxidation or functionalization treatments introduce additional oxygen- or nitrogen-containing groups that promote metal complexation. Reported adsorption capacities for modified SCGs frequently exceed 100 mg/g under favorable conditions. However, the extent of improvement depends strongly on the modification method and reaction parameters, making generalization difficult without considering specific experimental setups.

SCG-derived biochars typically exhibit improved adsorption performance compared with raw SCGs due to increased surface area, developed porosity, and enhanced structural stability. Nevertheless, their performance is not solely governed by surface area. In many cases, biochars produced at lower pyrolysis temperatures retain a higher density of oxygen-containing functional groups, which can enhance metal ion complexation. As a result, such materials may exhibit comparable or, in some cases, higher adsorption capacities for specific metal ions, despite their lower surface area as reported in several studies [58,97]. This highlights the importance of balancing pore development and surface chemistry.

Composite materials represent the most advanced class of SCG-based adsorbents, often achieving the highest reported adsorption capacities (in some cases exceeding 300 mg/g). These materials benefit from synergistic effects between the SCG matrix and incorporated components such as metal oxides or polymers.

**Figure 4:**
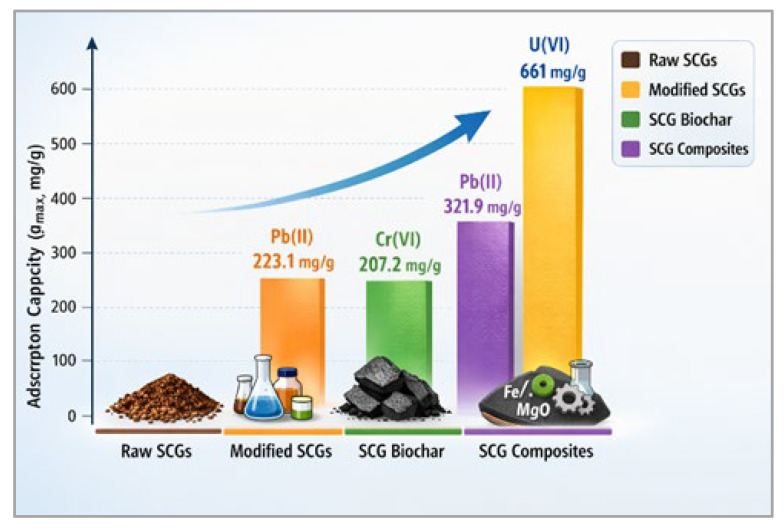
Comparison of maximum adsorption capacities reported for SCG-based adsorbents toward different metal ions.

To illustrate the resulting variability, the maximum adsorption capacities (q_max_) of selected SCG-based materials are compared in Figure 4. It should be noted that this comparison is intended to illustrate general trends rather than provide a strict ranking of material performance, due to differences in experimental conditions across studies. The values of adsorption capacities differ substantially across material types. Raw SCGs exhibit the lowest performance, while chemically modified and composite materials generally achieve higher capacities. SCG-derived biochars display intermediate behavior, with performance strongly dependent on pyrolysis conditions and activation methods.

It should also be noted that adsorption capacities reported for single-metal systems are generally higher than those obtained in multi-metal systems. Therefore, performance data obtained under single-solute conditions may overestimate the effectiveness of SCG-based adsorbents in practical applications.

The important limitation in the literature is the incomplete reporting of experimental parameters. In several studies summarized in Table 2, key variables such as pH or adsorbent dosage were not specified, which significantly limits the comparability of the results. This lack of standardized reporting represents a major barrier to systematic evaluation of adsorption performance.

In addition to equilibrium capacity, adsorption kinetics should also be considered when evaluating material performance. Many studies report that the adsorption of metal ions onto SCG-based materials follows the Langmuir isotherm model, suggesting monolayer adsorption on relatively homogeneous surfaces. However, this conclusion is often based on fitting quality (R^2^ values) rather than mechanistic validation and should therefore be interpreted cautiously, particularly when comparing different material classes. Moreover, the calculated rate constants vary widely depending on experimental conditions, particularly initial metal concentration and adsorbent properties. Therefore, generalized statements about adsorption rates or the kinetic superiority of specific materials should be avoided unless supported by systematically comparable datasets.

While the above discussion focuses primarily on equilibrium adsorption capacity obtained from batch studies, it should be emphasized that such systems do not fully represent real treatment conditions. Experimental data from a fixed-bed column are more representative and help assess potential applicability at the industrial scale [73,85]. In such a system, the breakthrough curve, e.g., a plot of c/c_0_ vs. time or bed volume, is an important parameter that indicates when the column becomes saturated. For As(V) ions (1 mg/L) in a column packed with raw SCGs coated with PEI and Fe(III), the breakthrough point was reached after 110 min, whereas at the same flow rate of 10 mL/min and 1 mg/L metal dosage, the breakthrough point for Cu(II) ions was reached after 60 min [85].

To improve the reliability of cross-study comparisons, future research should adopt standardized experimental protocols and reporting practices, including consistent pH, initial concentration, adsorbent dosage ranges, and complete disclosure of experimental conditions.

#### Framework for Comparison of Adsorption Performance of SCG-Based Materials

As mentioned above, direct comparison of maximum adsorption capacities reported for metal ions using SCGs is often hindered by significant variations in experimental conditions and adsorbent properties. To address this limitation, a structured framework for the normalized comparison of adsorption performance is proposed, incorporating both process parameters and material characteristics.

Firstly, adsorption data should be compared within similar pH ranges, as pH strongly affects both metal speciation and the surface charge of the adsorbent. Whenever possible, q**_max_** values should be evaluated within a narrow pH window (e.g., 5 ± 0.5), or alternatively grouped into defined pH ranges to ensure meaningful comparison. Secondly, adsorbent dosage (g/L) must be reported and considered, since higher dosages can lead to lower apparent adsorption capacities due to partial underutilization of active sites. Thirdly, normalization with respect to the initial metal concentration (C_0_) is recommended. This can be achieved either by reporting removal efficiency or by introducing a normalized adsorption capacity (q* = q**_max_**/C_0_), which enables comparison across studies conducted under different concentration regimes.

Furthermore, the method of SCG preparation plays a crucial role in determining adsorption performance; thus, comparisons should be performed within clearly defined material categories. The normalized comparison attempts, based on Pb(II) adsorption under different experimental conditions, are presented in Table 3. The dataset is limited to studies reporting sufficiently detailed experimental parameters, which significantly reduces the number of comparable entries. Moreover, biochar materials derived from SCGs were excluded from this comparison. Their adsorption performance is strongly governed by pyrolysis conditions (e.g., temperature, heating rate, residence time, and atmosphere), which fundamentally alter material properties such as surface area, porosity, and functional groups.

Even this limited dataset in Table 3 demonstrates that normalization can reveal consistent performance trends that remain obscured in conventional comparisons. When normalized adsorption capacity is considered, differences between materials become more apparent, enabling a more meaningful comparison across studies. In the analyzed dataset, q* values range from 0.21 to 0.57, with chemically modified SCGs exhibiting approximately two- to threefold higher values than raw or minimally treated materials. This indicates that surface functionalization has a stronger impact on adsorption efficiency than variations in initial concentration alone, a trend that is less evident when only q**_max_** values are compared. This observation is in agreement with the broader trends summarized in Table 2.

At the same time, the analysis highlights a key limitation in the current literature: inconsistent reporting of experimental conditions frequently prevents quantitative cross-study comparison and precludes a full meta-analysis. To improve comparability, future studies should systematically report at least pH, initial concentration (C_0_), adsorbent dosage, and isotherm model parameters.

### 4.2. Adsorption Mechanisms

The adsorption of metal ions onto SCG-based materials occurs through several mechanisms, including electrostatic attraction, ion exchange, surface complexation, and physical adsorption. The main mechanism varies depending on the type of adsorbent, the solution’s pH, and the chemical properties of the metal ions.

Functional groups such as carboxyl, hydroxyl, and amine participate in metal ion complexation through coordination bonding. Electrostatic interactions play a crucial role when the solution pH exceeds the point of zero charge of the adsorbent surface, resulting in attraction between negatively charged functional groups and positively charged metal ions. For most metal ions, adsorption is most efficient between pH 4 and 6. Below 4, the high concentration of H^+^ ions competes with metal ions for active sites on the SCG surface. These groups undergo deprotonation as pH increases, and at higher pH, they become more negatively charged, thereby enhancing electrostatic attraction and complexation of positive metal ions. However, when pH rises above 6–7, metal ions may precipitate as hydroxides, potentially limiting the efficiency of the adsorption process. Chwastowski et al. [73] reported that altering the pH did not significantly affect the sorption properties of SCG-biochar, as the material has buffering properties. Cr(VI) removal is influenced by its speciation in the aqueous phase, where different ionic forms (e.g., HCrO_4_^−^, Cr_2_O_7_^2−^) prevail depending on the pH, as well as by changes in the adsorbent surface charge. Below pH 5, the protonated surface of the SCGs exerts a strong electrostatic attraction for HCrO_4_^−^ anions. At higher pH levels, the surface of coffee waste carries a negative charge, leading to electrostatic repulsion that greatly diminishes Cr(VI) adsorption. In some cases, the partial reduction may occur to Cr(III), which can subsequently be immobilized on the adsorbent surface [76]. Similar relations between adsorption efficiency and sample pH were observed for the utilization of SCG-based adsorbents for As(V) oxyanions [63].

Ion exchange is another important mechanism, particularly in chemically modified SCGs and biochars containing mineral components. In this process, metal ions from the solution replace exchangeable ions present on the adsorbent surface.

### 4.3. Thermodynamic and Kinetic Parameters

Thermodynamic parameters provide valuable insights into the energy changes occurring during adsorption. Positive ΔH° values indicate that the adsorption process is endothermic, while positive ΔS° values are typically associated with increased disorder at the solid–solution interface. Increasingly negative ΔG° values with rising temperature indicate that the sorption process is spontaneous, thermodynamically favorable, and more efficient at higher temperatures. Based on the cited studies reporting these parameters, it can be concluded that the removal and enrichment of the studied metal ions using SCG-based adsorbents is an endothermic, spontaneous, and thermodynamically favourable process.

The mass transfer of metal ions to sorbents involves several steps, including diffusion from the bulk solution to the external surface of the material (film diffusion), diffusion within the solid matrix (intraparticle diffusion), and chemical interactions between the ions and surface functional groups. Identifying the rate-limiting step is crucial for improving sorption performance. For instance, if intraparticle diffusion is the limiting factor, increasing the adsorbent’s porosity can accelerate the process [99]. Common kinetic models include the pseudo-first-order, pseudo-second-order, intraparticle diffusion, and Elovich models [14]. The selection of an appropriate model is typically based on the coefficient of determination (R^2^) obtained from fitting experimental data, as well as on the agreement between theoretical and experimentally determined qₑ values. A review of the literature indicates that the linear form of the pseudo-second-order kinetic model most consistently provides the best fit to the experimental data.

However, the calculated rate constants (k_2_) for metal adsorption onto SCG-based materials using this model depend on the initial metal concentration and the type of surface modification. For example, k_2_ values of 0.499, 0.149, 0.104, 0.029, and 0.005 g/mg·min were reported for Pb(II) adsorption onto raw SCGs at initial concentrations of 100, 125, 150, 200, and 300 mg/L, respectively [73]. Similar trends were observed for Mn(II) and Zn(II) adsorption. Chemical treatment of SCGs typically increases the adsorbent surface area and the number of functional groups, thereby enhancing the kinetic rate constants. Typical ranges of pseudo-second-order rate constants are approximately 0.005–0.5 g/mg·min for raw SCGs, 0.02–0.8 g/mg·min for chemically modified SCGs, and 0.001–0.05 g/mg·min for SCG-derived biochar. Adsorption rates often follow the order: Cd(II) ≈ Cu(II) > Zn(II) > Pb(II). The adsorption rate also increases with decreasing particle size [96].

Furthermore, in some papers, kinetic data have been analyzed using the intraparticle diffusion model proposed by Weber and Morris to elucidate the diffusion mechanism [99,100,101]. Although the regression lines were linear, they did not pass through the origin, indicating that intraparticle diffusion is not the sole rate-controlling step and that additional mechanisms contribute to the overall adsorption process.

### 4.4. Adsorption Selectivity and Competitive Systems

In practical water and wastewater treatment systems, multiple metal ions are typically present simultaneously, which introduces competitive interactions for available adsorption sites. Therefore, in addition to adsorption capacity, selectivity toward specific metal ions is a key parameter determining the practical applicability of SCG-based adsorbents.

A comparison of selected studies summarized in Table 2 indicates that adsorption capacities obtained in multi-metal systems are generally lower than those observed in single-metal systems. This reduction can be attributed to competition between ions for active sites, which limits the overall uptake of individual species. However, despite this competition, certain trends in adsorption selectivity can be identified.

In many studies, SCG-based adsorbents exhibit a preferential affinity toward Pb(II), followed by Cd(II), Cu(II), Ni(II), and Zn(II), although the exact order may vary depending on material type and experimental conditions. This trend is commonly attributed to differences in the physicochemical properties of the metal ions, including ionic radius, electronegativity, hydration energy, and charge density. Metal ions with lower hydration energy and larger ionic radius, such as Pb(II), are more readily adsorbed because they can more easily shed their hydration shell and interact with functional groups on the adsorbent surface. In contrast, smaller and more strongly hydrated ions, such as Ni(II) or Zn(II), typically exhibit lower adsorption affinity under comparable conditions. Furthermore, ions with higher electronegativity and polarizability tend to form stronger complexes with oxygen- and nitrogen-containing functional groups present on SCG-based materials.

The type of adsorbent modification also plays a crucial role in determining selectivity. For example, the introduction of nitrogen-containing functional groups (e.g., amine groups) enhances the affinity toward transition metal ions through coordination bonding. Similarly, sulfur-functionalized biochars exhibit increased selectivity toward soft metal ions such as Cd(II), Hg(II), and Pb(II), consistent with the hard and soft acids and bases theory. In composite materials, incorporated metal oxides or polymers can further tailor selectivity by introducing specific binding sites or electrostatic interactions.

It should be emphasized that direct comparison of selectivity trends across studies remains difficult due to differences in experimental conditions and reporting practices. In particular, variations in initial metal concentrations, molar ratios in multi-component systems, and a lack of detailed speciation analysis can significantly influence observed selectivity sequences. Therefore, reported selectivity orders should be interpreted as system-specific rather than universally applicable.

Overall, although SCG-based adsorbents demonstrate promising selectivity toward certain metal ions, especially Pb(II), a more systematic investigation under controlled and comparable conditions is required to fully understand and predict their behavior in complex, real-world systems.

### 4.5. Reusability and Regeneration of SCG-Based Adsorbents

The reusability of adsorbents is a key parameter determining their practical applicability in water treatment, as it directly influences process cost, sustainability, and waste generation. In the case of SCG-based materials, regeneration and reuse have been investigated in numerous studies; however, meaningful comparison of reported results remains challenging due to methodological inconsistencies and insufficient reporting of experimental conditions.

In well-designed adsorption–desorption studies, regeneration is typically performed using appropriate eluents, including mineral acids (e.g., HCl, HNO_3_), bases (e.g., NaOH), complexing agents like EDTA, or organic solvents. These agents promote desorption of metal ions through protonation of surface functional groups, ion exchange, or complexation mechanisms. After desorption, the adsorbent is generally washed, dried, and reused in subsequent cycles, enabling simultaneous evaluation of adsorption capacity, retention, and desorption efficiency.

However, a critical analysis of the literature reveals that regeneration procedures are often inadequately described, particularly with respect to eluent type and concentration, contact time, and solid-to-liquid ratio. In some cases, regeneration is limited to simple washing with distilled water, which is unlikely to ensure complete removal of adsorbed metal ions. Consequently, the observed decrease in adsorption capacity over successive cycles may result not only from structural or chemical degradation of the adsorbent but also from incomplete desorption.

**Figure 5:**
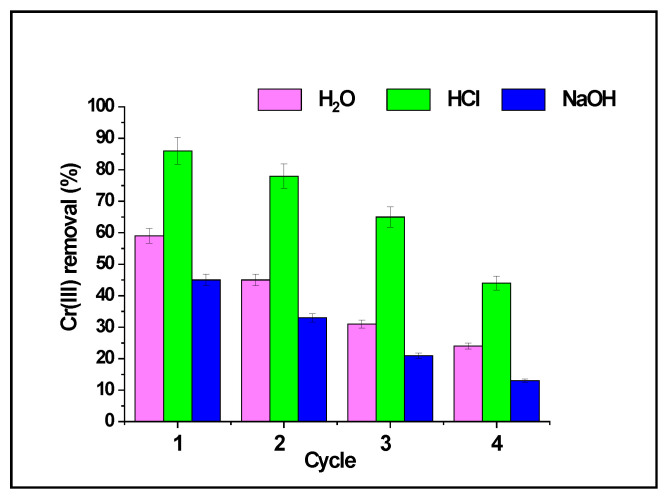
Reusability analysis of raw SCG for the adsorption of Cr(III). Based on data reported in [91].

Despite these limitations, approximate trends can be identified. SCG-based adsorbents, especially those subjected to chemical modification, typically retain 60–90% of their initial adsorption capacity after 3–5 cycles [25,43,68,73,91,102]. The gradual decline in adsorption efficiency over repeated cycles, as illustrated in Figure 5, is commonly attributed to partial loss of active sites, structural changes, or accumulation of residual adsorbates.

The efficiency of regeneration depends strongly on the nature of surface functional groups and the dominant adsorption mechanism. Adsorption governed by electrostatic interactions or ion exchange is generally more reversible, whereas processes involving strong surface complexation or precipitation tend to be less reversible. Chemical modification of SCGs, including activation and functionalization, can improve both adsorption capacity and regeneration performance by increasing the accessibility and reversibility of binding sites. In contrast, SCG-derived biochars may exhibit lower regeneration efficiency in some cases due to stronger binding of metal ions within the porous carbon matrix [66,72,81]. For example, Campbell et al. reported that NaOH was the most effective desorption agent for Cr(VI) from KOH-modified SCG biochar, although the desorption efficiency remained low (9.13% after 48 h), while acidic solutions showed negligible recovery [76].

Overall, although SCG-based adsorbents demonstrate promising reusability, the lack of standardized testing protocols and incomplete reporting of regeneration conditions significantly limit the comparability and reproducibility of published data. Future studies should adopt more rigorous and transparent methodologies, including detailed reporting of desorption conditions, evaluation of both adsorption and desorption efficiencies, and application of standardized multi-cycle testing protocols, to enable a more reliable assessment of long-term performance.

### 4.6. Comparison of SCG-Based Adsorbents with Alternative Materials for Pb(II) Removal

To better assess the practical applicability of SCG-based adsorbents, their performance should be evaluated against other commonly reported materials. The comparison presented in Table 4 for Pb(II) adsorption is based on general performance trends reported under broadly comparable conditions, rather than strict one-to-one quantitative equivalence.

SCG-based materials demonstrate adsorption capacities that are comparable to or, in some cases, higher than those of other lignocellulosic waste-derived adsorbents such as rice husk, sawdust, or banana peel biochar. These materials typically exhibit capacities in the range of 20–120 mg/g, depending on modification and processing conditions. Among natural biosorbents, chitosan-based materials often show relatively high affinity toward Pb(II), with capacities up to approximately 100 mg/g, although their production costs are higher.

Commercial activated carbons and engineered polymer-based adsorbents generally achieve higher adsorption capacities (often exceeding 200 mg/g) and exhibit more consistent performance. However, these materials are associated with higher production costs, more complex synthesis procedures, and, in some cases, less sustainable production pathways.

The cost-effectiveness of wastewater treatment using SCG biochar was compared to that of commercial activated carbon [76]. The calculations were based on treating 1000 m^3^ of wastewater per day containing Cr(VI) at a concentration of 13 mg/L, which was reduced to the permissible limit of 0.1 mg/L. The treatment cost using the SCG biosorbent was approximately £0.88, while the cost using commercial activated carbon was £0.93. Although the difference in treatment cost is relatively small, commercial activated carbon remains more expensive. Moreover, it exhibits a lower adsorption capacity (21.9 mg/g) and lower removal efficiency compared to SCG-biochar activated with KOH (207 mg/g). Furthermore, Mukherjee et al. evaluated the process economics of SCG biochar production (slow pyrolysis and CO_2_ activation) [121]. They found that its price (USD 0.15–0.21/kg, depending on the utilization of flue gas from the pyrolysis process) was lower than that of commercially available activated carbon (USD 0.45/kg). It is worth noting that the price of activated carbon varies across countries. Additionally, the produced activated carbon can be used for several applications, including wastewater treatment, catalyst supports, and energy applications, each of which requires specific properties that influence the final market price [122].

Thus, from an economic perspective, SCG-derived adsorbents offer a competitive advantage due to their low cost and potential for large-scale valorization of waste biomass. Reported cost analyses indicate that SCG biochar can be produced at significantly lower cost than commercial activated carbon, while achieving comparable or even superior adsorption performance under certain conditions. The combination of lower material cost, waste valorization potential, and sufficient adsorption efficiency makes SCG-based materials attractive for sustainable water treatment applications.

## 5. Conclusions

The findings of this review confirm that spent coffee grounds (SCGs), as an abundant lignocellulosic residue from the food industry, represent a promising resource within the circular bioeconomy, including reducing waste, reusing materials and recycling resources. Their valorization as adsorbent materials enables the conversion of a widely available waste stream into value-added products for the removal of metal ions from aqueous systems.

Both raw and modified SCGs exhibit notable adsorption performance, with chemical modification and carbonization significantly enhancing their capacity and applicability. In many cases, SCG-based materials achieve efficiencies comparable to other low-cost biosorbents, supporting their potential as sustainable alternatives to conventional adsorbents. However, inconsistencies in experimental conditions and insufficient reporting of key parameters limit comparability and reproducibility across studies. The lack of standardized methodologies remains a major barrier to evaluating long-term performance and practical applicability.

Despite these challenges, SCGs offer clear advantages in terms of availability, cost-effectiveness, and environmental impact. Future research should focus on standardizing testing protocols, improving understanding of adsorption mechanisms, and evaluating performance under realistic conditions, including complex wastewater matrices and scale-up scenarios. Strengthening collaboration with coffee industry stakeholders may further support the integration of SCG-based materials into circular economy frameworks.

To support practical selection of SCG-based adsorbents, a decision tree (Figure S1 in Supplementary Materials) was developed based on reported adsorption capacities, regeneration efficiency, and material characteristics. The tree guides users from the target metal ion and required adsorption performance to the most suitable type of SCG-based adsorbent: raw SCGs for low-capacity applications, chemically modified SCGs for moderate capacities and enhanced selectivity, and biochar or composite materials for high-capacity or specialized applications. Additional branches account for regeneration needs and functionalization requirements, providing a structured framework for material selection in practical water treatment scenarios.

Overall, SCG-based adsorbents represent a sustainable and scalable solution for metal ion removal, offering a unique balance between performance, cost-efficiency, and environmental impact, and thus hold strong potential for real-world water treatment applications.

## Figures and Tables

**Table 1:** Representative pyrolysis conditions for SCG-derived biochar used in metal ion removal.

Pyrolysis Conditions	Activation/ Modification	Surface Area (m^2^/g)	Target Metal Ions	Remarks	Ref.
500 °C for 2 h, heating rate 10 °C/min, with N_2_	n.r.	11.0	Sr(II)	Unmodified SCGs-biochar removed Sr(II) to a much greater extent than activated carbon.	[70]
500 °C for 2 h, heating rate 10 °C/min	n.r.	40.10	Ag(I)	99.9% silver ion removal efficiency from a 5 mg/L solution.	[71]
550 °C for 1.5 h, heating rate 10 °C/min with N_2_ in muffle or tube furnace	n.r.	3.29 (tube) 124.26 (furnace)	Pb(II), Ni(II)	Pyrolysis in a muffle furnace gave an adsorbent with a larger pore size (3.42 nm) than that from the tube furnace (0.015 nm).	[72]
700 °C for 3 h with CO_2_, fluidized bed reactor	n.r.	6.8	Cd(II), Mn(II), Pb(II)	Adsorption capacity order: Pb(II) < Cd(II) ~ Mn(II)	[73]
600 °C for 4 h	0.1 M HCl	n.r.	Pb(II)	55% of removal efficiency after 2 h from a 197 mg/L solution	[74]
426 °C for 93 min, heating rate 5 °C/min, with N_2_	H_3_PO_4_	n.r.	Cr(VI)	Preliminary hydrothermal carbonization (180 °C, 2 h) enhances the material’s properties.	[75]
400 °C for 2 h, heating rate 5 K/min, with N_2_	KOH	827.9	Cr(VI)	Increasing the KOH mass and pyrolysis time results in larger pore sizes.	[76]
800 °C for 2 h, heating rate 5 °C/min	2 M KOH	87.47	Cu(II) Pb(II), Cd(II), Cr(III)	Adsorption capacity order at pH 6: Cr(III) < Cu(II) < Cd(II) < Pb(II)	[77]
600 °C for 2 h	2% H_3_PO_4_, 2 M KOH	365.4 349.0	Li(I)	H_3_PO_4_-activated biochar exhibited a higher Li(I) adsorption capacity than the KOH-activated counterpart.	[78]
600 °C for 1 h, heating rate 10 °C/min	30% H_2_O_2_	n.r.	Cr(VI)	Reduction to Cr(III). The analogous reaction Mn(VII) → Mn(II) was observed.	[79]
700 °C, heating rate 10 °C/min, with CO_2_ or N_2_	FeCl_3_	468.4	As(V)	The surface area of biochar increased by ~70 times during pyrolysis with CO_2_ compared to N_2_.	[65]
400 °C for 1 h, heating rate 10 °C/min	FeSO_4_, FeCl_3_, 3 M KOH	360	Cd(II)	Removal of Cd(II) decreased with increasing pyrolysis temperature from ~97% to ~76%.	[80]
650 °C for 2 h with N_2_	FeCl_2/_FeCl_3_	62.75	Pb(II), Cu(II), Cd(II)	Porous, honeycomb-like structure resulting from the loading of iron nanoparticles.	[62]
Calcination at 140 °C (limited amount of air)	H_3_PO_4_, mercaptoacetic acid	781	Cd(II)	Reduction and deposition of Cd on the surface under the action of sulfoxide.	[65]
600 °C for 4 h	0.1 M KOH, arginine	301.68	Pb(II)	Decrease in BET surface area and pore volume after modification.	[68]

n.r.—not reported.

**Table 2:** Adsorption of metal ions onto SCG-based materials reported recently in the literature.

Metal Ions	Sorbent	Dose (g/L)	pH	Time (h)		q_max_ (mg/g)		Desorption/ Regeneration	Ref.
As(V)	SCG-Fe-biochar (N_2_) (CO_2_)	1	4.5–5	3		13.1 8.9		n.r.	[63]
Cd(II)	Raw SCGs	2.5	6	2		19.3		n.r.	[90]
	KMnO_4_ modified SCGs	1	n.r.	1		49.5		1 M HNO_3_	[51]
Cr(VI)	Raw SCGs	2.5	4	1.5		42.9		0.5 M HCl	[91]
	H_3_PO_4_-activated SCG-biochar	2	2	24		n.r.		n.r.	[75]
	HNO_3_-activated SCG-biochar	2.5	2	3		207.2		0.1 M NaOH	[76]
Cu(II)	Raw SCGs	n.r.	4.5	1		25.3		10% HCl	[92]
	Calcium alginate-SCGs	1	4	3		29.3		n.r.	[47]
Fe(III)	HNO_3_-modified SCGs	0.3	4	1		0.47		n.r.	[93]
Hg(II)	Raw SCGs	5	n.r.	4		0.06		n.r.	[94]
Li(I)	KOH-activated SCG-biochar H_3_PO_4_-activated SCG-biochar	0.5	n.r.	2		80.7 88.5		n.r.	[78]
Ni(II)	SCG-Fe-biochar with chitosan	1	6	6		108.70		0.1 M HCl	[84]
Pb(II)	Raw SCGs	0.1	n.r.	3		2.5		n.r.	[95]
	Fe_3_O_4_/PVA/SCGs	1.2	5	24		56.9		n.r.	[87]
	SCG-biochar	0.08	4	10		67.0		1M HCl	[72]
	Arginine-modified SCGs-biochar	0.1	n.r.	1.5		223.1		70% ethanol	[68]
	MgO-Fe-SCG-biochar	0.1	5	0.42		321.9		n.r.	[88]
Sr(II)	SCG-biochar	0.5	5	4		51.81		n.r.	[70]
U(VI)	SCGs-polyacrylic acid	1	3	1		661.0		0.1 M HNO_3_	[86]
***Multi-element mixtures***									
**Metal Ions**	**Sorbent**		**Dose (g/L)**	**pH**	**Time (h)**		q_max_ ** (mg/g)		**Ref.**
As(V), Cu(II)	PEI/Fe-coated SCGs		0.5	7.1	1		83.3; 200.1		[85]
Cu(II), Zn(II)	Raw SCGs		4	5	3		1.61; 4.52		[25]
Cu(II), Pb(II), Cd(II)	Fe-SCG-biochar		1.0–1.2	5	4		88.1; 103.9; 63.7		[62]
Cd(II), Mn(II), Pb(II)	SCG-biochar		2	7	0.5		13.6; 13.0; 11.0		[73]
Pb(II), Cu(II), Zn(II), Ni(II)	Raw SCGs		1	5.5	3		35.2; 11.4; 7.84; 5.82		[96]
Cu(II), Pb(II), Cd(II), Cr(III)	KOH-modified SCG-biochar		2	6	6		75.02; 229.67; 45.17; 51.68		[77]

PEI—polyethylenimine; PVA—polyvinyl alcohol; n.r.—not reported; ** Values correspond to ions listed in the same order. Note: Several studies did not report key experimental parameters such as pH, which significantly limits direct comparison of adsorption capacities.

**Table 3:** Examples of normalized comparisons of Pb(II) adsorption onto SCG-based materials.

Pretreatment	pH	C_0_ (mg/L)	Dosage (g/L)	q_max_ (mg/g)	q* = q_max_/C_0_	Ref.
Raw SCGs	n.r.	10	0.1	2.5	0.25	[95]
SCGs washed with acidified water	4.5	100	1	21.2	0.21	[98]
Fe_3_O_4_/PVA-modified SCGs	5	100	1.2	57	0.57	[94]

n.r.—not reported.

**Table 4:** Comparison of SCG-based and selected alternative adsorbents for Pb(II) removal.

Adsorbent	Modification	q_max_ (mg/g)	Key Advantages	Limitations	Ref.
Raw SCGs	none	5–100	Low cost, abundant, no processing	Lower capacity vs. engineered adsorbents	[73,96,101,103]
Chemically modified	Acid/base treated	20–120	More functional groups	Huge variability depending on treatment	[73,97,104,105]
SCG-biochar	Thermal	50–90	High surface area	Lower functional group density	[62,72,73,106]
SCG-biochar	Thermal + modification	50–320	Improved capacity, high surface area	Regeneration issues	[62,74,88,106,107]
Commercial activated carbon	Activated/modified	50–250	High efficiency	More expensive	[108,109,110,111]
Banana peel biochar	Thermal + KOH	43	Improved capacity	Difficulties in separation from treated water	[112]
Rice husk	Untreated /modified	20–120	Widely available, cheap	Variable composition	[113,114]
Sawdust	Modified	10–100	Low cost	Slower kinetics	[115,116]
Chitosan-based adsorbent	Cross-linked	70–100	High affinity Selectivity	Higher cost	[117,118,119]
Dry chamomile flowers	Base treatment	30	Low cost, natural	Limited data available	[120]

## Data Availability

No new data were created or analyzed in this study. Data sharing is not applicable to this article.

## Supplementary Materials

The following supporting information can be downloaded at: https://www.mdpi.com/article/10.3390/ma19091720/s1, Figure S1: Decision tree for selection of SCG-based adsorbents.
