# Supplementary figures and images for: Spent Coffee Grounds as an Adsorbent Material for Metal Ions
Source: Materials (Basel). 2026 Apr 23;19(9):1720. doi: 10.3390/ma19091720 (PMC13165226; doi:10.3390/ma19091720)

### Supplementary material

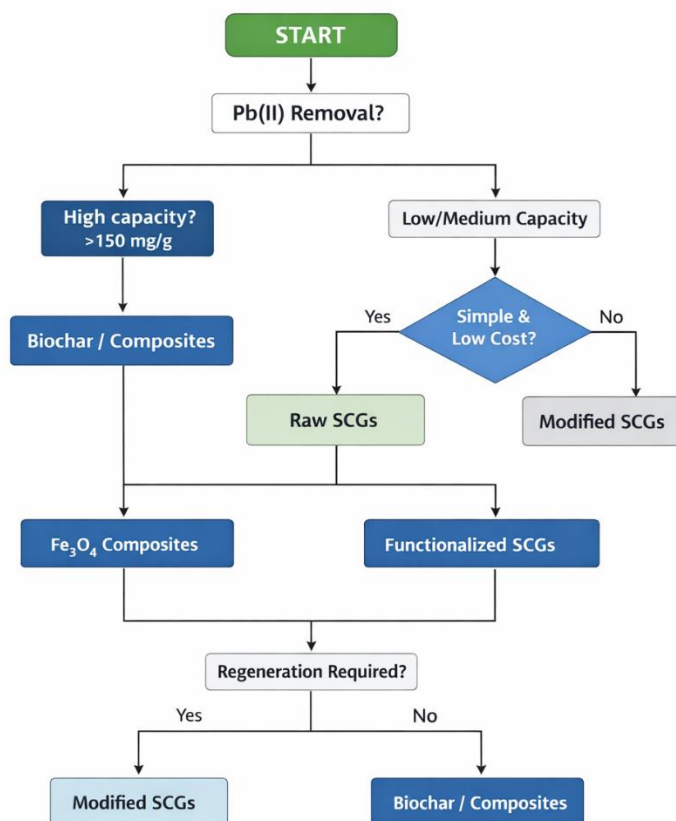

Supplement: Supplementary file 1 [file materials-19-01720-s001.zip › materials-4254945-supplementary.pdf]
